# Predicting the Risk of Mortality in Children using a Fuzzy-Probabilistic Hybrid Model

**DOI:** 10.1155/2022/7740785

**Published:** 2022-03-03

**Authors:** Corsino Rey, Juan Mayordomo-Colunga, Roberts Gobergs, Reinis Balmaks, Ana Vivanco-Allende, Andrés Concha, Alberto Medina, Ana Colubi, Gil González-Rodríguez

**Affiliations:** ^1^Department of Pediatrics, University of Oviedo, Oviedo, Spain; ^2^Department of Pediatrics, Hospital Universitario Central de Asturias, Oviedo, Spain; ^3^Instituto de Investigación Sanitaria del Principado de Asturias, Oviedo, Spain; ^4^Primary Care Interventions to Prevent Maternal and Child Chronic Diseases of Perinatal and Developmental OriginNetwork (RICORS), RD21/0012/0020, Instituto de Salud Carlos III, Madrid, Spain; ^5^Centro de Investigación Biomédica En Red-Enfermedades Respiratorias, Instituto de Salud Carlos III, Madrid, Spain; ^6^Department of Pediatrics, Riga Stradins University, Children's Clinical University Hospital, Riga, Latvia; ^7^University of Giessen, Giessen, Germany; ^8^Department of Statistics/Indurot, University of Oviedo, Oviedo, Spain

## Abstract

**Introduction:**

The mortality risk in children admitted to Pediatric Intensive Care Units (PICU) is usually estimated by means of validated scales, which only include objective data among their items. Human perceptions may also add relevant information to prognosticate the risk of death, and the tool to use this subjective data is fuzzy logic. The objective of our study was to develop a mathematical model to predict mortality risk based on the subjective perception of PICU staff and to evaluate its accuracy compared to validated scales.

**Methods:**

A prospective observational study in two PICUs (one in Spain and another in Latvia) was performed. Children were consecutively included regardless of the cause of admission along a two-year period. A fuzzy set program was developed for the PICU staff to record the subjective assessment of the patients' mortality risk expressed through a short range and a long range, both between 0% and 100%. Pediatric Index of Mortality 2 (PIM2) and Therapeutic Intervention Scoring System 28 (TISS28) were also prospectively calculated for each patient. Subjective and objective predictions were compared using the logistic regression analysis. To assess the prognostication ability of the models a stratified *B*-random *K*-fold cross-validation was performed.

**Results:**

Five hundred ninety-nine patients were included, 308 in Spain (293 survivors, 15 nonsurvivors) and 291 in Latvia (282 survivors, 9 nonsurvivors). The best logistic classification model for subjective information was the one based on MID (midpoint of the short range), whereas objective information was the one based on PIM2. Mortality estimation performance was 86.3% for PIM2, 92.6% for MID, and the combination of MID and PIM2 reached 96.4%.

**Conclusions:**

Subjective assessment was as useful as validated scales to estimate the risk of mortality. A hybrid model including fuzzy information and probabilistic scales (PIM2) seems to increase the accuracy of prognosticating mortality in PICU.

## 1. Introduction

There are numerous tools to employ when attempting to predict the outcome in critically ill children. They have been developed to predict the risk of death for patients while being admitted to pediatric intensive care units (PICUs). The most used scales are the various versions of Pediatric Risk of Mortality (PRISM) and Pediatric Index of Mortality (PIM). These scales were designed to estimate the mortality risk depending on clinical signs and routine analysis [1–5]. Other classification systems such as the therapeutic intervention scoring system (TISS) [6, 7] are based on therapeutic interventions, estimating severity based on the number and complexity of procedures performed on the patient. Clinicians use scoring systems because they believe that these models are more accurate than their own individual judgments [8].

In recent years, the advantages of modeling human perceptions or expert opinions have been performed through what is known as diffuse sets or fuzzy data. Fuzzy data are functional data with values on the scale [0,1], which help to represent the continuous nature of opinions (from completely agree to completely disagree) [9, 10]. Fuzzy logic is able to deal with imprecise and uncertain information, and this has been proposed to be useful in clinical decision making, considering that symptoms and diseases are “fuzzy” in nature. Furthermore, it has been suggested that fuzzy logic may offer complementarity with probability statistics [11].

The main goal of this study was to develop statistical methods to predict the risk of mortality of children admitted to PICU based on the subjective perception of the PICU staff (fuzzy data). As a secondary objective, we aimed to compare the performance of subjective staff estimation with a scale of risk of mortality (PIM2) and a scale of therapeutic interventions (TISS28).

## 2. Methods

We designed a prospective observational study set in two PICUs of University Hospitals from Spain (Oviedo) and Latvia (Riga). The study protocol was approved by the Hospital Ethics Committee of both participating hospitals, providing a waiver of consent for study participation. The protocol followed the Declaration of Helsinki principles. Data were recorded from January 2017 to December 2018.

### 2.1. Staff's Predictions

**Figure 1:**
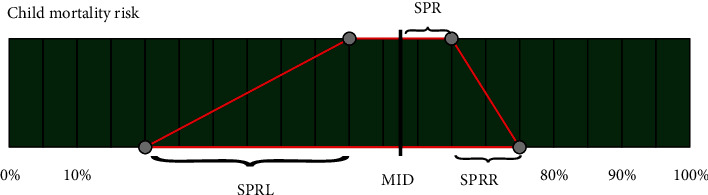
Trapezoid (red lines) created with the short range (upper) and the long range (lower) of the subjective evaluations performed by the staff (physicians and nurses). Four indexes were considered: MID: midpoint of the short range, SPR: radius of the short range, SPRL: distance from minimum point of the long range and minimum point of the short range, and SPRR: distance from maximum point of the long range and maximum point of the short range.

Data from physicians (attendings and residents) and nurses were obtained after the morning and afternoon rounds. Each subjective evaluation of the “child mortality risk” was recorded by means of a fuzzy set. PICU staff made their risk assessment for mortality utilizing a short and long range, both between 0% and 100%, during the first 20 hours after admission. Both ranges created a trapezoid (Figure 1) that has been parameterized by means of 4 indexes (MID: midpoint of the short range, SPR: radius of the short range, SPRL: distance from the minimum point of the long range and minimum point of the short range, and SPRR: distance from the maximum point of the long range and maximum point of the short range). These 4 indexes characterized the subjective mortality risk fuzzy set for each patient. The different subjective evaluations received by each patient were averaged to obtain a single subjective mortality risk per patient.

### 2.2. Study Population

All children admitted to PICU for more than 24 hours during the study period were eligible. The Spanish PICU dataset included 308 patients (139 female), whereas the Latvian dataset comprised 291 patients (132 females). Final outcomes during PICU admission included 15 deaths (4.9%) in Spanish patients and 9 deaths (3.1%) in Latvian patients. Age, sex, and diagnosis were prospectively recorded. PIM2 and TISS28 were the scales included in both PICUs' electronic recording systems. PIM2 was calculated at admission and TISS28 during the first 24 hours after admission, as it was the normal clinical practice [12]. PIM2 and TISS28 had been previously validated in Oviedo's PICU [1, 7].

The distribution of the number of subjective evaluations received by each patient is summarized in Table 1. Most patients received 1 to 3 subjective evaluations, varying the number of evaluations per patient around 2.85 in Oviedo and 2.65 in Riga (mean value). A total of 877 and 777 subjective evaluations were carried out in Spain and Latvia, respectively.

### 2.3. Ethics

The study protocol was approved by both Ethics Committees of the participating hospitals (Comité de Ética de la Investigación del Principado de Asturias and Riga Stradina Universitate Etikas Komiteja), providing waiver of consent for study participation.

### 2.4. Statistical Analysis

Data are presented as means and standard deviations (SD), medians and interquartile ranges (IQR), and numbers and proportions where appropriate. Statistical significance was set at *p* < 0.05.

To assess the ability to discriminate between survivors and nonsurvivors, a logistic regression model based on PIM2, TISS28, and subjective indexes was performed.

To estimate the predictive performance of the models, a stratified *B*-random *K*-fold cross-validation was performed. Considering that the outcome variable distribution is extremely asymmetric (survival rate was nearly 96%), balancing for nonsurvivor and survivor groups was required so that comparisons could be done. An approximation of 70% preset estimation accuracy of mortality was chosen. Variability was controlled by performing a large number of replications (*B* = 1000), and *K* = 4 − folds so that each fold comprised at least 2 deaths in the stratified scheme both for Oviedo and Riga datasets (and consequently for the combined dataset). Comparisons were performed based on the univariate Wald test as well as on the likelihood ratio test for nested models.

## 3. Results

### 3.1. Study Population

Baseline demographic, diagnostic, and scores of severity of the patients are shown in Table 2.

### 3.2. Logistic Model Based on Subjective and Objective Indexes

A logistic regression set was performed using four parameterized subjective indexes, obtained as described in Figure 1. As depicted in Table 3, the univariate Wald test showed that MID was the only significant subjective index to predict mortality in the Oviedo dataset, while in Riga, MID and SPRR were significant. Using these 2 variables, we then applied likelihood ratio tests for all nested models (Table 4). MID was found to be useful in mortality prediction, while SPRR was discarded as *p* value was > 0.05 when comparing the MID vs. MID+SPRR model. Thus, the best logistic classification model based on subjective information was the one based on MID.

Regarding objective indexes, PIM2 showed to be useful to predict mortality, whereas TISS28 was not informative in both datasets according to the Wald test (Table 3). This finding was corroborated by the likelihood ratio tests for the corresponding nested models (Table 4). It should be noted that the likelihood ratio test suggested that TISS28 was useful for the Oviedo and total datasets, contrary to the Wald test. However, adding TISS28 to the PIM2 model did not add any value to the model (*p* > 0.05). Therefore, the best logistic classification model based on objective information was the one based on PIM2.

Finally, the likelihood ratio test was also used to assess whether a hybrid model composed of MID and PIM2 could outperform the prediction ability of each single variable. The PIM2+MID model showed to be superior to PIM2 and MID models in Oviedo and total datasets, meaning that subjective and objective information were complementary to discriminate between survivors and nonsurvivors. However, this finding was not replicated in Riga's dataset (Table 4). When comparing the hybrid model with MID, *p* value was > 0.05, suggesting that the objective information was not able to improve the prediction ability of subjective information. On the contrary, the combined MID+PIM2 model improved the one based only on PIM2 (*p* < 0.0001). Therefore, in Riga's dataset, subjective information improved the model based on objective data.

### 3.3. Mathematical Validation of Predictive Performance of the Models

**Figure 2:**
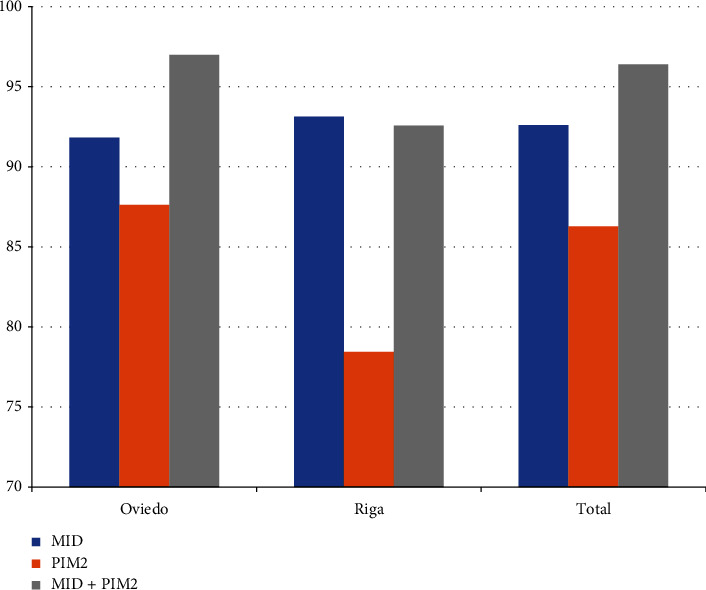
Prediction of survival accuracy for the Oviedo, Riga, and whole dataset using subjective (MID) and objective (PIM2) estimations, and the combination of both. Note that these estimations were performed using an approximation of 70% preset prediction of mortality accuracy (see Table 5). MID: logistic model using MID as explanatory variable; PIM2: logistic model using PIM2 as explanatory variable. MID+PIM2: logistic model using MID and PIM2 as explanatory variables.

The prognostication ability of the models was estimated by means of a stratified *B*-random *K*-fold cross-validation.  Table 5 and Figure 2 show the predictive performance of the subjective model (MID) vs. the objective model (PIM2) vs. the hybrid model (MID+PIM2). Pointwise estimation showed a better accuracy of the model based on subjective information (MID) than the one based on objective information (PIM2) to discriminate between survivors and nonsurvivors. In the Oviedo dataset, the accuracy to predict survival was 87.6% for PIM2, 91.8% for MID, and 97.0% for the hybrid model. However, in Riga, these percentages were similar for MID and MID+PIM2 (93.1% and 92.5%, respectively) and lower for PIM2 (78.4%). Regarding the overall sample, the results obtained when analyzing the Oviedo dataset were replicated.

## 4. Discussion

Our study investigated the performance of fuzzy data collection of perceptions of medical and nurse PICU staff for the prediction of mortality in critically ill children with different underlying conditions. We have found that subjective information from health care providers is useful to assess the risk of mortality in PICUs. Furthermore, the use of a hybrid model composed of fuzzy and objective data showed a very good ability to discriminate between PICU survivors and nonsurvivors. To our knowledge, this is the first prospective study in critically ill children which uses diffuse sets or fuzzy data to model expert opinions as outcome predictors.

Medical thinking is a clear example of vagueness, and fuzzy sets are employed to represent imprecise information. A fuzzy dataset was employed to collect the subjective information assessing the risk of mortality. A short range represented the most plausible probability including the likelihood of nonsurvival, and a long range was used to depict the almost certain inclusion of the risk of mortality, both ranging from 0 to 100%. The trapezoid created with these two ranges offers different measures to estimate the prediction accuracy (Figure 1). The midpoint of the short range (MID) was found to be the best classification model based on subjective information. It is well known that staff may be more likely to make mortality predictions on a continuous scale [13]. Although for obtaining MID only the short range is necessary, the use of short and long intervals makes data collection more comfortable for health care providers. Marcin et al. [14, 15] reported that the higher the level of certainty associated with mortality prediction, the more accurate the prediction. Therefore, subjective mortality prediction may be dependent on the level of confidence in the prognosis. We hypothesized that the addition of a long range in the subjective evaluation would increase the level of staffs' confidence in their prognosis, making the short-range estimation more accurate.

Regarding the objective information provided by the two validated scores, we found that PIM2 was useful to predict mortality, whereas TISS28 was not useful in the Riga PICU and did not improve PIM2 prediction in the Oviedo PICU. This is an unsurprising result because PIM2 has been developed to predict the risk of death for critically ill children [1, 4, 5], whereas TISS28 measures the intensity of therapeutic interventions and therefore the nursing workload in everyday practice [6]. Estimation of mortality risk by TISS28 is a surrogate of the number and complexity of therapeutic interventions assuming a higher risk of death if more procedures and more complex interventions are required.

Several studies have been published [16–22] comparing staff predictions with scoring systems to predict mortality in adult intensive care units (ICUs). However, we only found two studies [14, 15] about the prognostication of mortality risk by health care providers conducted in the PICU, and the objective was not to compare staff vs. validated scale predictions. In a systematic review including 12 observational studies, Sinuff et al. [13] concluded that ICU physicians discriminate between survivors and nonsurvivors more accurately than do scoring systems in the first 24 hours of ICU admission. Our results are in agreement with these studies. Moreover, in the total sample and the Oviedo PICU, MID and PIM2 were both useful and complementary. Scholz et al. [23] also reported, in a study performed in two German ICUS, that prognoses made by physicians were superior to objective models. They considered that physicians did not limit their prognosis to objective factors, but they based their opinions on experience and individual observations. Our results coincide with this hypothesis. Subjective predictions provided by MID could be based on different information from the one used to calculate PIM2. While PIM2 is calculated from various coefficients corresponding to closed categories (for example, high risk vs. low risk diagnosis), PICU staff can consider comorbidities or chronic illness beyond the categories used by PIM2 and provide physicians and nurses with further information about the actual patient status.

Some studies performed in the adult populations [13] and in premature neonates [24, 25] describe the “self-fulfilling prophecy” arguing that more accurate predictions by staff could reflect a lack of support for children who would otherwise have a reasonable likelihood of a favorable outcome. It has been reported that obstetricians and pediatricians who underestimate neonatal survival are less likely to provide beneficial therapy [26]. We do not consider this explanation as determinant of our results because the subjective predictions were made during the first 20 hours after admission, before decisions to withdraw any kind of life support were considered.

There are significant differences comparing data from Oviedo and Riga. Children from Oviedo were younger and diagnosed at admission were mainly medical, whereas almost half of the patients from Riga were postoperative. As a consequence, TISS28 was higher in the Riga PICU, reflecting that surgical patients require more therapeutic interventions from nursing staff than medical patients. On the other hand, PIM2 was lower in Riga (*p* = 0.059), suggesting the admission of less severe patients. Although there are differences in patient characteristics and the cultural background of PICU staff which could be different in both countries, the subjective ability to predict the risk of mortality was very similar (Table 5). On the other hand, PIM2 was useful in both PICUs, but when combined with MID, PIM2 increased the predictive performance only in the Oviedo PICU. Several reasons may explain why the PIM2 score was lower and had lower predictive ability in Riga. One is related to the type of admission; while almost half of the patients in Riga were surgical, and most admissions in Oviedo were emergent. The latter are scored higher in PIM2. Similarly, patients with noninvasive ventilation score as “mechanical ventilation,” and many patients from Oviedo were receiving this type of respiratory support.

Since we began this study, Jacobs et al. [27] have recently reported that the most recently developed and validated version of the PIM score, PIM3, showed good mortality prediction accuracy. PICU care changes over time. Thus, we need to make sure that the risk of mortality scoring systems reflects contemporary PICU care paradigms [28]. Future multicenter studies should include PIM3 as the objective scale.

Our study has several limitations. First, this was a two-center study that includes different groups of critically ill children. Although both PICUs reported the same results from the subjective information provided by MID, the prediction of adverse outcome could be different in other PICUs if the population was different. This fact limits the generalizability of the results and establishes a need for multicentre international studies to achieve a better understanding of such outcome predictors. Second, the small sample size with a low mortality rate limits statistical analysis. Third, we used PIM2 and not the most recent version of PIM because the former was available in both PICUs. We cannot exclude some differences in our results if PIM3 had been employed.

This study has several strengths. First, no previous pediatric studies have subjective information collected through a fuzzy dataset with a short and long range to predict the risk of mortality. Second, it is prospective in design, and hence, we obtained detailed data collection. Finally, we used bootstrapping [29, 30] techniques with resampling to overcome the limitations posed by a relatively small sample size, especially in the nonsurvivor population.

## 5. Conclusions

In summary, a hybrid model composed of subjective and objective information provided by MID and PIM2 had a good ability to discriminate between survivors and nonsurvivors in the PICU. Subjective prediction of mortality obtained through a fuzzy dataset seems to be an interesting and useful tool to increase the accuracy of the likelihood of survival prognostication.

## Figures and Tables

**Table 1:** Distribution of patients according to the number of evaluations performed by the staff (physicians and nurses).

Number of evaluations	1	2	3	4	5	6	7	8	≥9
Number of patients (Spain)	73	83	71	43	19	7	4	3	5
Number of patients (Latvia)	23	109	115	41	3	2	—	—	—

**Table 2:** Demographic, diagnostic, and mortality score data.

	Oviedo	Riga	*p* value	Total
Patients (survivors/nonsurvivors)	308 (293/15)	291 (282/9)	0.2681	599 (575/24)
Age (years) (mean; SD)	5.29; 5.10	7.40; 6.79	***<**0.001*	6.32; 6.08
Sex (female %)	45.13%	45.36%	0.9548	45.24%
Diagnosis (number; %)				599; 100%
Respiratory	137; 44.48%	19; 6.53%	**<** *0.001*	156; 26.04%
Circulatory	15; 4.87%	12; 4.12%	0.6389	27; 4.51%
Infectious	19; 6.17%	23; 7.90%	0.3973	42; 7.01%
Neurological	40; 12.99%	32; 11.00%	0.4628	72; 12.02%
Traumatic	22; 7.14%	28; 9.62%	0.2730	50; 8.35%
Oncohematological	8; 2.60%	2; 0.69%	0.0772	10; 1.67%
Metabolic-renal	13; 4.22%	21; 7.22%	0.1160	34; 5.68%
Postoperative	48; 15.58%	134; 46.05%	**<** *0.001*	182; 30.38%
Others	6; 1.95%	20; 6.87%	**<** *0.01*	26; 4.34%
MID (mean; SD)	9.82; 17.74	9.73; 13.94	0.9565	9.77; 16.02%
MID survivors	7.55; 12.29	8.51; 10.82	0.306	8.00; 11.59%
MID nonsurvivors	54.57; 37.83	50.00; 33.22	0.6691	52.09; 36.31%
PIM2 (%) (mean; SD)	4.44; 13.61	2.35; 5.58	0.059	3.15; 9.82%
PIM2 survivors	2.29; 6.96	1.97; 4.440	0.5016	27.46; 32.61%
PIM2 nonsurvivors	35.33; 37.60	14.33; 15.8	0.0838	2.14; 5.51%
TISS28 (absolute value) (mean; SD)	17.21; 7.54	25.01; 8.98	**<** *0.001*	20.96; 9.09%
TISS28 survivors	16.60; 6.64	24.83; 8.90	**<** *0.001*	29.00; 11.18%
TISS28 nonsurvivors	28.07; 12.00	30.55; 9.45	0.5947	20.64; 8.83%

MID: midpoint of the trapezoid top interval. PIM2: Pediatric Index of Mortality 2. SD: standard deviation. TISS28: Therapeutic Intervention Scoring System 28.

**Table 3:** Coefficients obtained in the univariate Wald test logistic model based on subjective and objective indexes.

	Oviedo estimate/SE/*p* value	Riga estimate/SE/*p* value	Total estimate/SE/*p* value
MID	0.049/0.013/**<***0.001*	0.078/0.018/**<***0.001*	0.041/0.009/**<***0.001*
SPR	-0.012/0.070/0.87	0.028/0.082/0.73	-0.008/0.039/0.84
SPRL	-0.059/0.053/0.27	0.112/0.059/0.06	0.013/0.027/0.64
SPRR	0.032/0.038/0.41	0.051/0.022/**<***0.02*	0.017/0.012/0.15
PIM2	0.065/0.020/**<***0.01*	0.104/0.039/*<0.01*	0.079/0.017/**<***0.001*
TISS28	0.036/0.037/0.33	0.017/0.039/0.65	0.021/0.024/0.38

SE: standard error; MID: midpoint of the trapezoid top interval; SPR: radius of the 1-cut; SPRL: distance from infimum of 0-cut to infimum of 1-cut; SPRR: distance from supremum of 1-cut to supremum of 0-cut; PIM2: Pediatric Index of Mortality 2; TISS28: Therapeutic Intervention Scoring System 28.

**Table 4:** *p* values obtained for the likelihood ratio between nested models.

	Oviedo	Riga	Total
*Subjective information*			
MID vs. NULL	**<0.001**	**<0.001**	**<0.001**
SPRR vs. NULL	0.671	0.106	0.139
MID+SPRR vs. NULL	**<0.001**	**<0.001**	**<0.001**
MID+SPRR vs. MID	0.658	0.077	0.192

*Objective information*			
PIM2 vs. NULL	**<0.001**	**<0.001**	**<0.001**
TISS28 vs. NULL	**<0.001**	0.080	**<0.001**
PIM2+TISS28 vs. NULL	**<0.001**	**0.001**	**<0.001**
PIM2+TISS28 vs. PIM2	0.353	0.663	0.390
PIM2+TISS28 vs. TISS28	**<0.001**	**0.001**	**<0.001**

*Combined information*			
MID+PIM2 vs. NULL	**<0.001**	**<0.001**	**<0.001**
MID+PIM2 vs. MID	**<0.001**	0.173	**<0.001**
MID+PIM2 vs. PIM2	**<0.001**	**<0.001**	**<0.001**

NULL: the null hypothesis. MID: logistic model using MID as explanatory variable. SPRR: logistic model using SPRR as explanatory variable. PIM2: logistic model using PIM2 as explanatory variable. TISS28: logistic model using TISS28 as explanatory variable. MID+SPRR: logistic model using MID and SPRR as explanatory variables. PIM2+TISS28: logistic model using PIM2 and TISS28 as explanatory variables. MID+PIM2: logistic model using MID and PIM2 as explanatory variables.

**Table 5:** Mortality prediction of subjective (MID) and objective (PIM2) indexes for the Oviedo, Riga, and joint dataset. Estimations were performed using an approximation of 70% preset prediction of mortality accuracy.

	Oviedo		Riga		Total	
MID	Real mortality		Real mortality		Real mortality	
Predicted mortality	Survivors	Nonsurvivors	Survivors	Nonsurvivors	Survivors	Nonsurvivors
Survivors	*91.82%*	30.09%	*93.14%*	29.64%	*92.61%*	30.25%
Nonsurvivors	8.18%	*69.91%*	6.86%	*70.42%*	7.40%	*69.75%*

PIM2	Real mortality		Real mortality		Real mortality	
Predicted mortality	Survivors	Nonsurvivors	Survivors	Nonsurvivors	Survivors	Nonsurvivors
Survivors	*87.62%*	30.17%	*78.44%*	29.58%	*86.28%*	30.52%
Nonsurvivors	12.38%	*69.83%*	21.56%	*70.42%*	13.72%	*69.48%*

MID+PIM2	Real mortality		Real mortality		Real mortality	
Predicted mortality	Survivors	Nonsurvivors	Survivors	Nonsurvivors	Survivors	Nonsurvivors
Survivors	*96.99%*	29.23%	*92.57%*	30.12%	*96.40%*	29.88%
Nonsurvivors	3.01%	*70.77%*	7.43%	*69.88%*	3.60%	*70.12%*

## Data Availability

Data are available on reasonable request through the corresponding author.

## Additional Points

*Key Points.* (i) Fuzzy logic is able to deal with imprecise and uncertain information, and this has been proposed to be useful in clinical decision making, considering that symptoms and diseases are “fuzzy” in nature. (ii) The present study showed that subjective (fuzzy) information was useful in assessing the risk of mortality of children admitted to two different pediatric intensive care units (PICUs). (iii) Subjective assessment of the risk of mortality was as accurate as validated scales. (iv) A hybrid model composed of subjective and objective information had a good ability to discriminate between survivors and nonsurvivors in the PICU.
